# High-throughput density-functional perturbation theory phonons for inorganic materials

**DOI:** 10.1038/sdata.2018.65

**Published:** 2018-05-01

**Authors:** Guido Petretto, Shyam Dwaraknath, Henrique P.C. Miranda, Donald Winston, Matteo Giantomassi, Michiel J. van Setten, Xavier Gonze, Kristin A. Persson, Geoffroy Hautier, Gian-Marco Rignanese

**Affiliations:** 1 Institute of Condensed Matter and Nanoscience (IMCN), Université catholique de Louvain, B-1348 Louvain-la-neuve, Belgium; 2 Lawrence Berkeley National Laboratory, Berkeley, California 94720, USA; 3 Department of Materials Science and Engineering, University of California, Berkeley, California 94720, USA

**Keywords:** Density functional theory, Semiconductors, Theory and computation

## Abstract

The knowledge of the vibrational properties of a material is of key importance to understand physical phenomena such as thermal conductivity, superconductivity, and ferroelectricity among others. However, detailed experimental phonon spectra are available only for a limited number of materials, which hinders the large-scale analysis of vibrational properties and their derived quantities. In this work, we perform *ab initio* calculations of the full phonon dispersion and vibrational density of states for 1521 semiconductor compounds in the harmonic approximation based on density functional perturbation theory. The data is collected along with derived dielectric and thermodynamic properties. We present the procedure used to obtain the results, the details of the provided database and a validation based on the comparison with experimental data.

## Background & Summary

The phonon spectrum of a material describes the dynamics of its constituent atoms in the harmonic approximation, in the framework of the long-established theory of lattice vibrations^1,2^. The details of the lattice dynamics are of key importance as many properties can not be explained by static models. A simple example is the set of thermal properties extracted from the phonon density of states (DOS), such as the vibrational contribution to the entropy of the system and the heat capacity^3–5^. But the vibrational properties of crystalline solids are also needed to investigate a number of other materials features, such as the thermal conductivity^6–8^, the conventional phonon-mediated superconductivity^9–11^ and the ferroelectric and ferroelastic transitions^12–15^. Additionally, they provide information for the investigation of the phase stability of compounds^16^ through the inspection of imaginary phonon modes and for the interpretation of Raman experimental spectra^17^.

Experimental phonon band structures are available only for a limited set of compounds and, in some cases, only for specific points of the Brillouin zone. Density functional theory (DFT) offers the possibility to obtain the vibrational properties of materials using frozen-phonon^18^ or molecular dynamics^19,20^. Alternatively, density functional perturbation theory (DFPT) is an accurate and efficient tool to calculate the lattice dynamics^21^. Given the requirements of these simulations, it is just recently, with the increase of computational power and the diffusion of high-throughput (HT) frameworks^22–24^, that handling and analysing large numbers of phonon calculations has been made possible.

**Figure 1:**
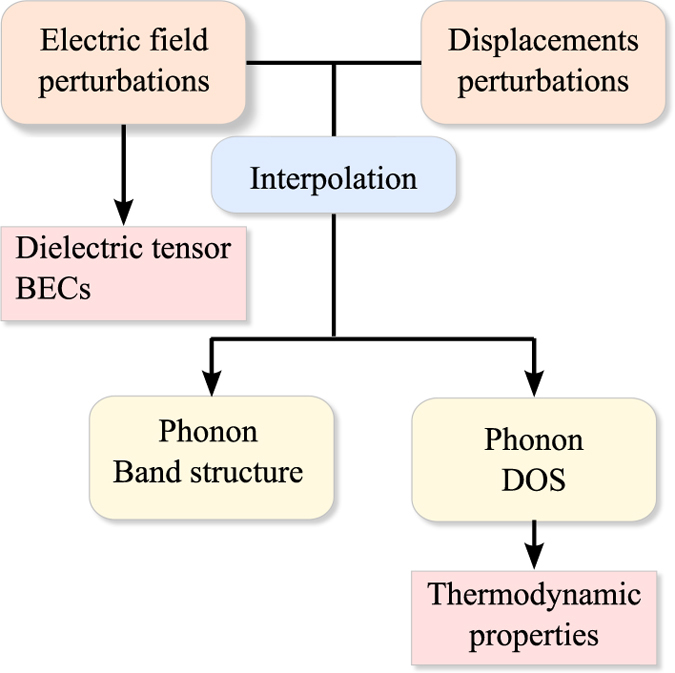
Schematic overview of the quantities calculated in this work. The first boxes indicate the perturbations computed in the framework of DFPT.

Standard studies reporting phonon calculations based on DFT usually consider few materials in selected phases. More recently efforts have been devoted to evaluate the phonon band structures for a large number of compounds (Atsushi Togo's phonon database http://phonondb.mtl.kyoto-u.ac.jp and ref. 25). In the present work, we report the full phonon band structures and derived quantities for 1521 semiconducting inorganic crystals, obtained following the procedure and the approximations detailed in our previous work^26^. The phonons are related to the second order derivatives of the energies with respect to the atomic displacements. However, to obtain the correct behavior for long range interactions in the case of polar materials, the coupling between the displacements and the electric field^27^ must also be considered. The latter is related to the mixed second order derivatives of the energy with respect to the electric field and atomic displacements. These derivatives, as well as those purely with respect to the electric field, can be efficiently calculated in the framework of DFPT. They give access to the Born effective charges (BECs) and the dielectric tensors, respectively. Here, we provide this full set of second order derivatives in an open database. The schematic overview of available properties and the procedure to obtain them is outlined in Fig. 1.

This derivatives database offers the possibility to analyze the lattice dynamics of a large number of compounds, generated with uniform approximations and under a validated procedure. These results are part of the Materials Project^28^ (MP) which uses HT methods to predict material properties for the discovery and design of new compounds.

The remainder of the paper is organized as follows. First, we define the phonon properties calculated and the procedure employed to obtain them. We then describe the structure of the data present in the database and give a graphical representation. Finally, we provide a validation of the results based on a comparison with experimental data.

## Methods

### Methodology and definitions

Most of the methodology and notations follow closely ref. 29. For a generic point **q** in the Brillouin zone the phonon frequencies *ω*_***q***,*m*_ and eigenvectors *U*_*m*_(***q**κ*′*β*) can be obtained by solving of the generalized eigenvalue problem

(1)∑κ′βC˜κα,κ′β(q)Um(qκ′β)=Mκωq,m2Um(qκα),

where *κ* labels the atoms in the cell, *α* and *β* are cartesian coordinates and ${\tilde{C}}_{\kappa \alpha ,\kappa \prime \beta }(q)$ are the interatomic force constants in reciprocal space, which are related to the second derivatives of the energy with respect to atomic displacements. These values have been obtained by performing a Fourier interpolation of those calculated on a regular grid of *q*-points obtained with DFPT.

To correctly describe the limit **q**→0 for polar materials, with the splitting between longitudinal and transverse optical modes (LO and TO, respectively) the dipole-dipole interaction has been taken into account. This requires the knowledge of both the BEC and the dielectric tensors^29^. The BEC tensor can be linked either to the change of polarisation P induced by the periodic displacement *τ*_*κα*_, or to the force *F*_*κ*,*α*_ induced on atom *κ* by an electric field $E_{\beta }$

(2)Zκ,βα*=Ω0∂P∂τκα=∂Fκ,α∂Eβ=∂2E∂τκα∂Eβ,

where *E* is the total energy, and is an observable quantity.

In the theoretical formulation, the interatomic force constants $\tilde{C}$ and BEC tensors Z* must satisfy a series of sum rules. The first, following from the invariance of total energy with respect to translations (known as acoustic sum rule, ASR)

(3)∑κC˜κα,κ′β(q=0)=0,

implies that the acoustic modes at Γ are identically zero. The second rule

(4)∑κZκ,βα*=0,

guarantees that the charge neutrality is fulfilled at the level of the BECs (CNSR). Both are imposed during the interpolation process to improve the results, but the actual deviation from the exact condition can be used to estimate the degree of convergence of the calculation (see below).

The second derivatives with respect to the electric field E allow one to also obtain the dielectric permittivity tensor resulting from the electronic polarization, usually noted as $\varepsilon_{\alpha \beta }^{\infty }$. This, together with the values of the phonon frequencies at the center of the Brillouin zone *ω*^Γ^_*m*_ and the oscillator strength tensor *f*_*m*,*αβ*_, gives the static dielectric tensor

(5)εαβ0=εαβ∞+4π∑mfm,αβ2(ωmΓ)2.

Given the phonon DOS *g*(*ω*)

(6)g(ω)=13nN∑q,lδ(ω−ω(q,l)),

where *n* is the number of atoms per unit cell and *N* is the number of unit cells, several thermodynamic quantities can be obtained in the harmonic approximation: the Helmholtz free energy Δ*F*, the phonon contribution to the internal energy Δ*E*_ph_, the constant-volume specific heat *C*_*v*_ and the entropy *S*. The explicit expressions are given by^3^:

(7)ΔF=3nNkBT∫0ωLln(2sinhℏω2kBT)g(ω)dω

(8)ΔEph=3nNℏ2∫0ωLωcoth(ℏω2kBT)g(ω)dω

(9)Cv=3nNkB∫0ωL(ℏω2kBT)2csch2(ℏω2kBT)g(ω)dω

(10)S=3nNkB∫0ωL(ℏω2kBTcoth(ℏω2kBT)−ln(2sinhℏω2kBT))g(ω)dω,

where *k*_*B*_ is the Boltzmann constant and *ω*_*L*_ is the largest phonon frequency.

Notice that in cases where imaginary frequencies are present in the system, these thermodynamic properties are ill defined and will not be calculated.

All the DFT and DFPT calculations presented in this work are performed with the ABINIT software package^30–32^. The PBEsol^33^ semilocal generalized gradient approximation for the exchange-correlation functional (XC), that has proven to provide accurate phonon frequencies compared to experimental data^34^, has been used for all the simulations. Norm-conserving pseudopotentials^35^, generated with the appropriate XC functional, are taken for all the elements from the pseudopotentials table PseudoDojo version 0.3 (ref. 36). The plane wave cutoff is chosen based on the hardest element for each compound, according to the values suggested in the PseudoDojo table.

The Brillouin zone has been sampled using equivalent *k*-point and *q*-point grids that respect the symmetries of the crystal and with a density of approximately 1500 points per reciprocal atom, as suggested in ref. 26. The *q*-point grid is always Γ-centered. All the structures are relaxed with strict convergence criteria, i.e. until all the forces on the atoms are below 10^−6^ Ha/Bohr and the stresses are below 10^−4^ Ha/Bohr^3^.

For all the materials, the primitive cells and the band structures are defined according to the conventions of Setyawan and Curtarolo^37^.

### Numerical precision estimation

We have identified a set of indicators that can give hints about the level of numerical precision of our results. The main ones are the aforementioned breaking of the acoustic and charge neutrality sum rules. While these properties are explicitly imposed, the breaking can be usually reduced by increasing the plane wave cutoff. This suggest that a large breaking could signal a lack of convergence with respect to this parameter.

We have also observed^26^ that the presence of small negative frequencies for the acoustic phonon frequencies in the close proximity of the Γ point could be associated with poor choices of the *k* or *q* point grids. In particular, we have observed that these are hardly ever a signal of a real incommensurate instability.

Despite the presence of these indications of a possible lack of convergence, the results obtained from the calculations with the imposition of the sum rules can still be reliable or give rather accurate values away from problematic regions, providing useful information, especially for screening purposes over large sets of data. Potentially problematic calculations are thus included in the database along with the numerical value of the ASR and CNSR. Three flags will help quickly identify these cases. One is set if the largest acoustic mode at Γ is larger than 30 cm^−1^, when the ASR is not explicitly imposed. A second one signals that the value of

(11)maxα,β∑κZκ,βα*

is larger than 0.2, that corresponds to the breaking of the CNSR. The last one indicates the presence of negative frequencies just in the region 0<|***q***|<0.05 in fractional coordinates along the high symmetry lines. Materials with likely real instabilities, showing negative (imaginary) frequencies also beyond this limit, do not have this flag set.

### Workflow

**Figure 2:**
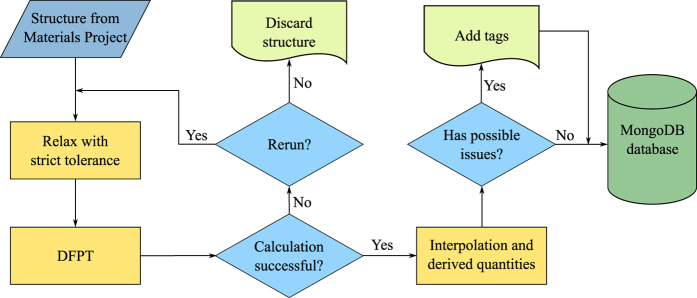
Workflow scheme used for calculating the phonon properties.

The workflow employed to handle our HT calculation is outlined in Fig. 2. The structures present in the MP database^28,38^ are taken as starting point, considering only semiconducting and insulating materials. Since these have been optimized within the projector augmented wave framework and for a different XC functional (PBE), we first perform a full relaxation of the system with strict convergence parameters. The following step consists in running the DFPT simulations to obtain the second derivatives of the energy with respect to the different perturbations considered. These calculations are carried out in parallel over all the perturbations and all the *q*-points. If the calculations are completed correctly, the set of derivatives is then used to generate the phonon band structure and DOS, along with the derived quantities, using a Fourier interpolation scheme. Unsuccessful calculations are analyzed and rerun if possible or discarded otherwise.

At this point the results undergo the controls defined in the previous section, i.e. the breaking of the ASR and CNSR and the presence of small negative frequencies close to Γ, and the corresponding flags are set in the record. A further flag is added if the band structure shows any negative frequency with absolute value larger than 5 cm^−1^, to signal the likely presence of an instability.

Finally, all the records are inserted into the MP database. From this they will be made available on the MP website and a JSON (JavaScript Object Notation) data document is generated for each record. A copy of all the JSON documents is available for download from the Figshare repository (Data Citation 1).

### Code availability

The open source code ABINIT^30–32^ is used throughout this work for calculations of phonon properties. ABINIT is distributed under the GNU General Public Licence. The workflows used to run the simulations are implemented using FireWorks as workflow manager^39^ (https://github.com/materialsproject/fireworks) and specific workflows are available in the Abiflows package (https://github.com/abinit/abiflows). The Pymatgen^40^ and AbiPy (https://github.com/abinit/abipy) python packages are used to generate inputs and analyze the results. Pymatgen is released under the MIT (Massachusetts Institute of Technology) License and is open source. AbiPy is released under the GNU GPL license. FireWorks is released under a modified BSD license.

## Data Records

The calculated phonon properties and derived quantities for 1521 materials are made available in this work. The materials include only inorganic solid semiconductors and insulators, with 1508 of these having less than 13 atomic sites per cell. The second order derivatives of the energy in the ABINIT derivative database file format (DDB) and the processed results in the JSON format can be downloaded from the Figshare repository (Data Citation 1). The data will additionally be made accessible through the Materials Project website (www.materialsproject.org) where we will provide static and interactive plots of the phonon dispersion.

### Second order derivatives of the total energy

The outputs of the DFPT calculation are the second order derivatives of the energies with respect to atomic displacements on a regular grid in the Brillouin zone and the second order derivatives of the energies with respect to static homogeneous electric field. These quantities are stored in the ABINIT DDB file format which is a human readable text format. A DDB file for each material is available in the Figshare repository (Data Citation 1).

### Processed data

The processed data for each of the calculated material is stored as a JSON document (Data Citation 1). JSON is a textual lightweight data-interchange format, that can be easily parsed by machines. It is built on two kinds of structures i) a collection of key/value pairs and ii) an ordered list of values. These structures can be nested. Each JSON file contains the data for a single material with the top level keys described in Table 1. The content of the second level is detailed in the Tables 2,3,4,5,6.

The metadata defined in Table 2 provides a description of the material and its characteristics, as well as details about the approximations employed in the calculation. The phonon properties are reported as described in Table 3. The phonon band structure is available for *n*_qpts_ *q*-points along the high-symmetry path, as defined in ref. 37, for each of the modes (*n*_modes_=3×*n*_sites_). The DOS is reported with two keys describing the list of frequencies and the corresponding values of the DOS. The thermodynamic properties obtained from the integration of the DOS are calculated on a uniformly spaced list of *n*_*T*_ temperatures with each property as a list of corresponding values as shown in Table 4. In the case where large negative frequencies are present in the material, the thermodynamic properties have not been calculated and the values corresponding to the *thermo* key at the top level (see Table 1) is empty. Dielectric properties and BECs are given according to the description provided in Table 5.

The estimations of the breaking of the sum rules are given, as defined in previous sections, in Tables 3 and 5. Two flags signal the cases where these values are considered large and the corresponding keys are given in Table 6, with two more flags concerning the presence of negative frequencies.

### Graphical representation of results

**Figure 3:**
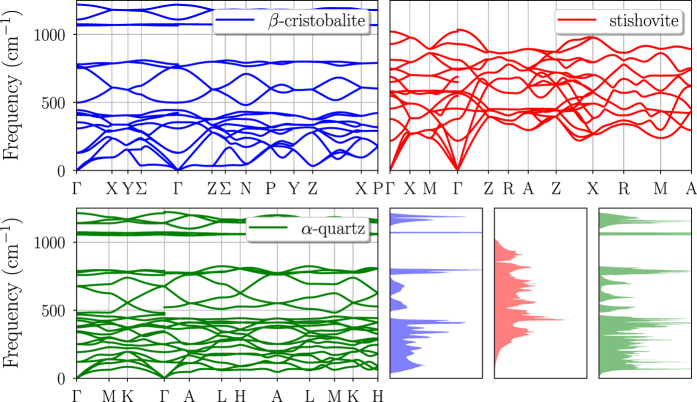
Comparison of phonon band structures and DOS for different phases of SiO_2_. The three phases are *β*-cristobalite (blue), stishovite (red) and *α*-quartz (green).

**Figure 4:**
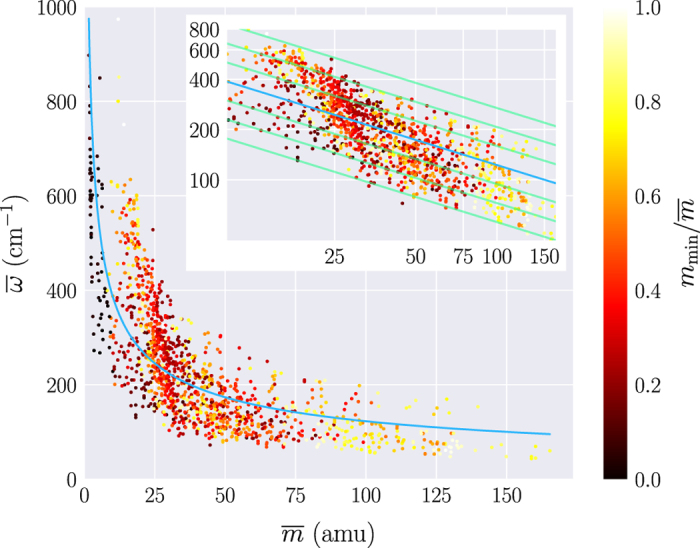
The average phonon frequency *ω*^−^ versus the average atomic mass *m*^−^. The color represents the minimum atomic mass of the system with respect to the average atomic mass *m*_min_/*m*^−^. The inset shows a zoom of the data in a log-log scale. The blue line represent a hyperbolic fit of the data, while green lines indicates hypothetical hyperbolic behavior with different constants. Only materials without negative frequencies have been considered.

Figures 3 and 4 are examples of graphical representation of the data stored in the database. The data reported under the *phonon* key of the JSON file (Table 3) can be used to plot the phonon band structure and DOS of each calculated material. An example showing the comparison of phonon band structures and DOS for three different phases of SiO_2_ (*β*-cristobalite, stishovite and *α*-quartz), is reported in Fig. 3.

Figure 4 illustrates the correlation between the average phonon frequency *ω*^−^, calculated as

(12)ω¯=∫ωg(ω)dω∫g(ω)dω,

and the average atomic mass of the compound, that we define as

(13)m¯=1n∑κMκ−2,

to better fit the values in equation (1). Only the cases where no negative frequencies are present in the phonon spectrum have been considered. As expected based on the relation between masses and frequencies given by equation (1), heavier elements are usually associated with lower average frequencies and the data follows the trend *ω*^−^~1/(*m*^−^)^1/2^. The data displays a spread around the hyperbolic fit, since the phonon frequencies are the outcome of the interplay of the whole set of interatomic force constants and of the different masses of the elements composing the material. It can be noticed that some trends can be recognized with respect to the masses of the components. Systems with non-uniform masses (identified by the small ratio *m*_min_/*m*^−^), tend to lay on a different hyperbolic curve with respect to more uniformly weighted systems.

## Technical Validation

The methodology used in this work and the choice of the sampling of the Brillouin zone have been analyzed in a previous study^26^. There, it has been shown that properly converged results can be obtained with a sampling of the Brillouin zone that respects the symmetry of the system and with a density of 1500 points per reciprocal atom, both for the *k*-point and *q*-point sampling. The results have been validated by comparing the sound velocities obtained from the phonon band structures with those calculated from the elastic tensors and checking the errors of the calculated vibrational entropy with respect to experimental data (see equation (10)). The tests showed a satisfactory accuracy, especially for screening purposes.

The pseudopotentials available in the PseudoDojo table^36^ have been evaluated by checking the Δ value with respect to the results obtained from all electron DFT codes^41,42^. However, since for the PBEsol functional these reference values do not exist, we used input parameters almost equivalent to those obtained for the PBE table to generate the PBEsol pseudopotentials. Further tests have been carried out on these new pseudopotentials to ensure a limited breaking of the ASR, exclude the presence of ghost states in the occupied and empty regions, and convergence of phonon modes at Γ. All these tests have been used to determine optimal values for the suggested energy cutoffs.

As a further validation, we present the comparison of the phonon frequencies at the Γ point calculated in this work with the available experimental data for 53 compounds.^34,43–70^ The majority of the materials considered are cubic systems with few atoms per supercells. However at least one material per crystal system is present in the test set, with system sizes up to 12 atoms per unit cell.

**Figure 5:**
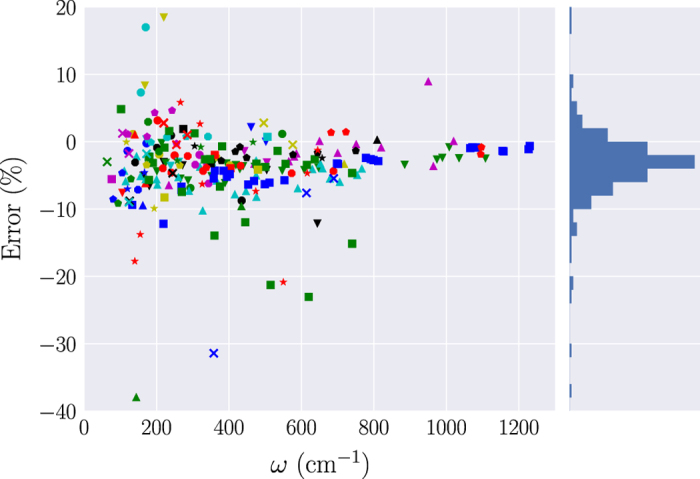
Relative error of selected calculated frequencies with respect to experimental data. The test set consists of the frequencies at the Γ point for which experimental values are available in literature. Each of the 53 considered materials is identified by a unique combination of symbol and color. The bar plot shows the distribution of the errors.

We considered all the frequencies at Γ for which the experimental data are available and matched them with the calculated values according to the symmetry of the modes. In Fig. 5 we report the relative errors of each of these frequencies with respect to the experimental data. With a mean relative error (MRE) over all the frequencies of −3.6%, it can clearly be seen that, on average, the simulations underestimate the values of the phonon frequencies. This underestimation, however, has been observed for different generalized gradient approximations (GGA) to the XC functional^34^ and is not limited to the case of the PBEsol employed in this work. Satisfactorily, almost all the errors are within 10%, with just 15 frequencies exceeding this threshold. In these cases, the calculated values are in good agreement with other simulations present in literature, showing that the origin of the disagreement does not lie in a lack of precision in our procedure. In particular, the worst examples in the set, with errors of −38% and −31.5%, belong to rock salt BaO (mp-1342) and to cubic ZrO_2_ (mp-1565), respectively. It has been shown^71^ that the large error in the TO mode of BaO is the result of the occurrence of strong anharmonicities, so that such frequencies cannot be accurately reproduced using the harmonic approximation. On the other hand, since cubic zirconia is unstable in normal conditions, the experimental values are obtained from yttria-stabilized samples. This is likely to perturb the phonon frequencies with respect to the ideal values.

With a mean absolute relative error (MARE) of 4.6%, we then conclude that our test set is in reasonably good agreement with the experimental data available.

**Figure 6:**
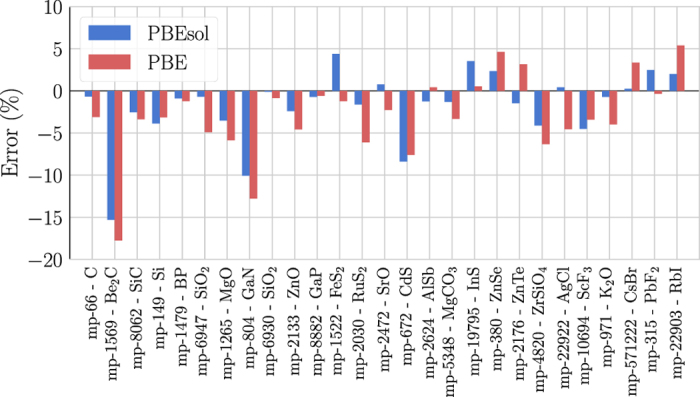
Relative errors on the calculated entropy *S* with respect to experimental data for PBEsol and PBE XC functionals. The entropy has been evaluated at room temperature (~300*K*, depending on experimental data available) neglecting the anharmonic effects. Each blue and red bar represents the error for a material for PBEsol and PBE functionals, respectively. The MARE is 2.97% for PBEsol and 4.25% for PBE.

Finally, in order to strengthen our choice of using the PBEsol XC functional, we evaluated the error of the values of the entropy (see equation (10)) at ~300*K* obtained in this work with respect to experimental data. These can be compared with the errors obtained previously using the Perdew-Burke-Ernzerhof (PBE)^72^ approximation in ref. 26. The relative errors with respect to experimentally measured data are shown for both the functionals in Fig. 6 for 27 compounds. As already remarked, the agreement is quite good, considering also that at room temperature the thermal expansion and the anharmonic effects, not included in our simulations, can already be playing a role.

While for a few materials the error obtained for the PBEsol values are larger than that coming from the PBE calculations, the former provide in general a better agreement, with a MARE over all the materials of 2.97% for PBEsol versus 4.25% for PBE. This analysis further confirms the higher accuracy of the PBEsol approximation with respect to PBE.

## Usage Notes

We present the processed phonon and dielectric properties of 1521 semiconducting materials. These can be used, for example, to identify trends in vibrational frequencies and thermodynamic properties. The data is provided in JSON files allowing to quickly extract information from the dataset.

If more detailed knowledge of specific quantities is required, we refer the users to the DDB files. These contain all the second order derivatives of the energies with respect to atomic perturbations on a set of points in the irreducible zone and the second order derivatives of the energies with respect to a static homogeneous electric field. These files can be processed using the *anaddb* code^31^ to extract several quantities. These include, (i) phonon frequencies at any point in the Brillouin zone, with and without the imposition of the sum rules discussed above, (ii) phonon band structures along custom paths in the Brillouin zone, (iii) interatomic force constants in real space (iv) projected phonon density of states. Ample documentation is available on the ABINIT website (https://www.abinit.org) and a python interface to run the postprocessing tool and plot the results is provided in the AbiPy package. Documentation for the usage of this python package is available online (http://pythonhosted.org/abipy, https://github.com/abinit/abitutorials/blob/master/abitutorials/ddb.ipynb). Example scripts are provided in the Figshare database (Data Citation 1).

## Additional information

**How to cite this article**: Petretto, G. et al. High-throughput density functional perturbation theory phonons for inorganic materials. *Sci. Data* 5:180065 doi: 10.1038/sdata.2018.65 (2018).

**Publisher’s note**: Springer Nature remains neutral with regard to jurisdictional claims in published maps and institutional affiliations.

## Supplementary Material



## Figures and Tables

**Table 1:** First level JSON keys. Each entry contains several key/value pairs at as values.

Key	Description
metadata	Metadata of the material (see Table 2)
phonon	Phonon properties (see Table 3)
thermo	Thermodynamic properties (see Table 4)
dielectric	Dielectric properties and BECs (see Table 5)
flags	Flags describing the calculation (see Table 6)

**Table 2:** Metadata.

Key	Datatype	Description
material_id	string	MP ID number
formula	string	Chemical formula
structure	string	Crystal structure in Crystallographic Information File (CIF) format
kpoints_grid	array	List of integers describing the regular grid of *k*-points
kpoints_shifts	array	List of shifts used in the ABINIT-specific format
qpoints_grid	array	List of integers describing the regular grid of *q*-points
cutoff	number	Plane wave cutoff (Ha)
pseudopotential_md5	array	List of MD5 hashes uniquely identifying the pseudopotentials
point_group	string	Point group in Hermann-Mauguin notation
space_group	number	Space group number as defined by the International Union of Crystallography
nsites	number	Number of atoms in the primitive cell

**Table 3:** Phonon properties.

Key	Units	Datatype	Shape	Description
ph_bandstructure	cm^−1^	array	*n*_qpts_×*n*_modes_	Phonon frequencies along high symmetry lines
qpts	−	array	*n*_qpts_×3	Fractional coordinates of the *q*-points along high symmetry lines
ph_dos	−	array	*n*_freqs_	DOS values
dos_frequencies	cm^−1^	array	*n*_freqs_	Frequencies corresponding to the DOS values
asr_breaking	cm^−1^	number	−	Maximum frequency of an acoustic mode at Γ (breaking of the ASR)

**Table 4:** Thermodynamic properties.

Key	Units	Datatype	Shape	Description
temperature	K	array	*n*_*T*_	Temperatures sampled for the thermodynamic properties
entropy	J/mol/K	array	*n*_*T*_	Values of the vibrational entropy
C_v	J/mol/K	array	*n*_*T*_	Values of the heat capacity
helmholtz_energy	J/mol	array	*n*_*T*_	Values of the Helmholtz free energy
phonon_energy	J/mol	array	*n*_*T*_	Values of the phonon contribution to the internal energy

**Table 5:** Dielectric properties and Born effective charges.

Key	Units	Datatype	Shape	Description
becs	*e*	array	*n*_sites_×3×3	Born effective charges
eps_electronic	−	array	3×3	Electronic contribution to the dielectric permittivity tensor
eps_total	−	array	3×3	Total dielectric permittivity tensor
cnsr_breaking	*e*	number	−	Maximum breaking of the CNSR

**Table 6:** Flags identifying possibly problematic materials.

Key	Datatype	Description
has_neg_fr	boolean	True if negative frequencies are present
large_asr_break	boolean	True if the breaking of ASR is greater than 30 cm^−1^
large_cnsr_break	boolean	True if the breaking of CNSR is greater than 0.2
small_q_neg_fr	boolean	True if negative frequencies are present only close to Γ
